# CXCL9, a promising biomarker in the diagnosis of chronic Q fever

**DOI:** 10.1186/s12879-017-2656-6

**Published:** 2017-08-09

**Authors:** Anne F. M. Jansen, Teske Schoffelen, Julien Textoris, Jean-Louis Mege, Marrigje Nabuurs-Franssen, Ruud P. H. Raijmakers, Mihai G. Netea, Leo A. B. Joosten, Chantal P. Bleeker-Rovers, Marcel van Deuren

**Affiliations:** 1 Department of Internal Medicine 463, Radboud center for Infectious Diseases (RCI), Radboud University Medical Center, P.O. Box 9101, 6500 HB, Nijmegen, The Netherlands; 2 Radboud Expert Center for Q fever, Radboud University Medical Center, P.O. Box 9101, 6500 HB, Nijmegen, The Netherlands; 3 Université Claude Bernard Lyon 1, Hospices Civils de Lyon, bioMérieux; EA7426 “Pathophysiology of injury induced immunosuppression (PI3)”, Hôpital E. Herriot, Lyon, France; 4 URMITE, Aix-Marseille University, Marseille, France; 5 Department of Medical Microbiology and Infectious Diseases, Canisius Wilhelmina Hospital, Nijmegen, The Netherlands

**Keywords:** Chronic Q fever, *Coxiella burnetii*, Chemokines, Biomarker, CXCL9

## Abstract

**Background:**

In the aftermath of the largest Q fever outbreak in the world, diagnosing the potentially lethal complication chronic Q fever remains challenging. PCR, *Coxiella burnetii* IgG phase I antibodies, CRP and ^18^F–FDG-PET/CT scan are used for diagnosis and monitoring in clinical practice. We aimed to identify and test biomarkers in order to improve discriminative power of the diagnostic tests and monitoring of chronic Q fever.

**Methods:**

We performed a transcriptome analysis on *C. burnetii* stimulated PBMCs of 4 healthy controls and 6 chronic Q fever patients and identified genes that were most differentially expressed. The gene products were determined using Luminex technology in whole blood samples stimulated with heat-killed *C. burnetii* and serum samples from chronic Q fever patients and control subjects.

**Results:**

Gene expression of the chemokines *CXCL9*, *CXCL10*, *CXCL11* and *CCL8* was strongly up-regulated in *C. burnetii* stimulated PBMCs of chronic Q fever patients, in contrast to healthy controls. In whole blood cultures of chronic Q fever patients, production of all four chemokines was increased upon *C. burnetii* stimulation, but also healthy controls and past Q fever individuals showed increased production of CXCL9, CXCL10 and CCL8. However, CXCL9 and CXCL11 production was significantly higher for chronic Q fever patients compared to past Q fever individuals. In addition, CXCL9 serum concentrations in chronic Q fever patients were higher than in past Q fever individuals.

**Conclusion:**

CXCL9 protein, measured in serum or as *C. burnetii* stimulated production, is a promising biomarker for the diagnosis of chronic Q fever.

**Electronic supplementary material:**

The online version of this article (doi:10.1186/s12879-017-2656-6) contains supplementary material, which is available to authorized users.

## Background

Q fever is a zoonosis caused by the intracellular bacterium *Coxiella burnetii*. Clinical presentation ranges from asymptomatic acute infection to pneumonia or hepatitis. One to 5 % of infected individuals develop chronic Q fever, characterized by a persistent infection of heart-valves, vascular aneurysms or prostheses. Chronic Q fever patients may present years after their initial infection with *C. burnetii*. If left untreated, this condition has a high mortality rate [1, 2]. To prevent irreparable damage, early diagnosis of chronic Q fever is essential [3]. Today, diagnosis of chronic Q fever depends on Polymerase Chain Reaction (PCR), measurement of anti-*C. burnetii* phase I IgG titers and imaging techniques [4–6]. Both laboratory techniques have drawbacks: PCR on blood has a low sensitivity and the cut-off values of anti-*C. burnetii* phase I to distinguish past from chronic infection are still debated and are either not sensitive or specific enough [7].

Even more challenging is the monitoring of chronic Q fever during treatment. Currently, monitoring relies on anti-*C. burnetii* phase I IgG titers, with a fourfold decrease or a drop below 1:800 as criteria to stop treatment [2]. In daily practice, titers hardly decrease and antibiotic therapy is continued for many years [8]. In addition, many clinicians measure C-reactive protein levels to monitor disease activity. Imaging by FDG-PET/CT scan is a promising tool in antimicrobial treatment decision making and its value in chronic Q fever is under investigation [5, 6]. The limitations of the current tests highlight the need for additional biomarkers in diagnosis and monitoring.

To assess new candidate biomarkers for the diagnosis of chronic Q fever and monitoring treatment, we performed a transcriptome analysis on *C. burnetii*-stimulated peripheral mononuclear cells of chronic Q fever patients. We validated the results in whole blood cultures and serum of patients with Q fever and control subjects.

## Methods

### Subjects

**Fig. 1:**
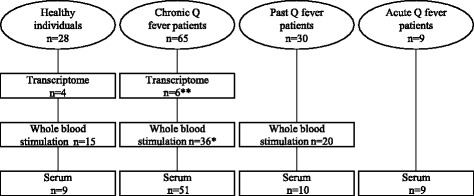
Flow chart of subject groups Total number of individuals in each group is depicted in the *circles*. The number of specimens per experiment of each group is described in the *squares*.*, **: for chronic Q fever patients, 6 patients that were included in the transcriptome analysis and 22 patients of whom ex-vivo whole blood stimulations were performed, also provided serum specimens

Four groups were included: healthy controls, patients with acute Q fever, patients with a history of cured acute Q fever (past Q fever), and patients with chronic Q fever (Fig. 1). Healthy controls (*n* = 28) were hospital personnel and students without a history of Q fever. Healthy controls with available serum samples (*n* = 9) were Q fever seronegative. Specimens of anonymous acute Q fever patients (*n* = 9), all PCR-positive for *C. burnetii* at the time of blood-drawing, were collected from left-over samples of patients identified between 2008 and 2009 in the Canisius Wilhelmina (CWZ) Hospital. Left-over serum specimens of past Q fever patients (*n* = 20), were also derived from the CWZ-hospital. Another 10 individuals with past Q fever, who provided heparin-anticoagulated blood, were recruited in the Q fever prevaccination screening program, described in detail by Schoffelen et al. [9]. All samples were *Coxiella burnetii* phase II IgG positive and had low phase I IgG titers, indicative for the absence of chronic Q fever. Chronic Q fever patients (*n* = 65) visited the outpatient clinic of the Radboud university medical center (*n* = 29) or the other participating hospitals: Catharina Hospital Eindhoven (*n* = 12), Elisabeth Hospital Tilburg (*n* = 9), CWZ-Hospital (*n* = 6), Elkerliek Hospital Helmond (*n* = 5) and Bernhoven Hospital Uden (*n* = 4). The chronic Q fever group comprised 47 proven and 18 probable chronic Q fever patients diagnosed according to the Dutch consensus guideline on chronic Q fever [4]. Thirty-seven patients presented with a vascular focus in an aneurysm or aortic prosthesis, 19 had valvular defects, 6 had both a vascular and valvular localization, and 3 patients were immunocomprised. All patients were on antibiotic treatment.

EDTA-anticoagulated blood for isolation of peripheral blood mononuclear cells (PBMCs) was collected from chronic Q fever patients and healthy controls. Heparin-anticoagulated blood for stimulation experiments was drawn from healthy controls, past Q fever and chronic Q fever patients.

### Peripheral blood mononuclear cells isolation and transcriptome analysis

PBMCs were isolated from blood centrifuged with Ficoll-Paque (GE Healthcare, Uppsala, Sweden) according to standardized procedures [10]. Cells were taken from the interphase layer, washed twice in Phosphate Buffered Saline, and counted in a Coulter Counter Z (Beckman Coulter, Fullerton, CA, USA). PBMCs were then incubated in flat-bottom 24-wells plates in 1 mL (10^7^cells/mL) and stimulated with heat-killed *C. burnetii* Nine Mile (NM) RSA493 phase I (kindly provided by dr HJ Roest, CVI, Lelystad) in 10^7^/mL at 37 °C for 8 h. RNA was extracted with the RNeasy Mini kit (Qiagen, Hilden, Germany). With the 2100 Bioanalyzer (Agilent Technologies, Massy, France) and the RNA 6000 Nano LabChip kit, the quality of the RNA was ensured. RNA quantity was determined with the Nanodrop (Thermo Scientific). Gene expression was analysed using Whole Human Genome 4x44K microarrays (Agilent Technologies, Massy, France) and One-color Microarray based Gene Expression Analysis kit, as previously described [11]. Gene expression data was analysed with R and Bioconductor software suite. Data was normalized with quantile normalization after being preprocessed and quality checked (Agi4x44PreProcess library). With the Limma library, differential gene expression was assessed. Data was compliant with the Minimun Information About a Microarray Experiment (MIAME) guidelines, accessible with number GSE66476 at the National Center for Biotechnology Information’s Gene Expression Omnibus, (www.ncbi.nlm.nih.gov/geo/).

### Whole blood stimulation experiments

Blood was drawn into 5 mL heparinized tubes. Subsequently 500 μL blood was incubated with either heat-killed *C. burnetii* NM in an end concentration of 10^7^/mL or with culture medium (negative control) [12]. After 24 h of incubation at 37 °C and 5% CO_2_, the supernatants were harvested and stored at -80 °C.

### Measurement of proteins and anti-*C. burnetii* serology

Interferon-γ (IFN-γ) production in stimulated whole blood supernatants was measured with commercially available enzyme-linked immunosorbent assay (ELISA) (Sanquin, Amsterdam, Netherlands) according to the manufacturer’s instructions. The CXCR3-chemokines and CCL8 were determined in stimulated whole blood supernatants and serum using Luminex technology (Bio-Rad, CA, USA). CRP from serum was measured with immunoturbidimetry (Architect, Siemens; and Cobas 6000, Roche) and anti-*C. burnetii* antibodies were determined in serum by indirect immunofluorescence measuring IgM and IgG against *C. burnetii* NM phase I and II a commercially available immunofluorescence assay (IFA, Focus Diagnostics Inc., Cypress, CA, USA).

### Statistical analysis

Data was analysed using GraphPad Prism and SPSS software programs and presented with medians and interquartile range (IQR) or minimum to maximum range (range) as cytokine and chemokine data is considered non-parametric. Differences between groups were assessed with the Kruskal Wallis test and completed with Dunn multiple comparison tests, a *p*-value of <0.05 was considered significant. Spearman’s rho was used for determining correlation. To assess the accuracy the proposed biomarkers, a receiver operator characteristic (ROC) curve was constructed and the area under the curve (AUC) was calculated. Whether there was a correlation of the biomarkers with time after start of treatment was determined per patient by a non-linear best fit regression model.

## Results

### Gene expression profiles reveal up-regulation of IFN-γ inducible genes in chronic Q fever

Transcriptional profiles of PBMCs of 4 healthy controls were compared to the PBMCs of 6 chronic Q fever patients that were in-vitro stimulated with heat-killed *C. burnetii.* To look for candidate biomarkers, genes were selected that were most differentially expressed in both groups. Four genes encoding for chemokines ended at the top of the list: CXCL9 also named Monokine Induced by IFN-γ (MIG), CXCL10 (IFN-γ Inducible Protein-10 (IP-10)), CXCL11 (IFN-γ T-cell Attractant Chemokine (I-TAC)), and CCL8 (Monocyte Chemotactic Protein (MCP-2)) (Table 1). CXCL9, CXCL10 and CXCL11 are ligands for CXCR3 and are therefore named CXCR3-chemokines.

**Table 1:** RNA expression in *C. burnetii* stimulated PBMCs

	*C. burnetii* stimulated PBMCs		
	Healthy controls	Chronic Q fever patients	Ratio patient/healthy control
CXCL11 / I-TAC	0.10	15.5	155
CXCL9 / MIG	0.45	62.5	139
CCL8 / MCP-2	0.50	46.3	93
CXCL10 / IP-10	0.20	16.5	83

RNA expression in C. burnetii stimulated PBMCs of healthy controls (*n* = 4) and chronic Q fever patients (*n* = 6). Median fold change from unstimulated condition is indicated. *Abbreviations*: *PBMCs* peripheral blood mononuclear cells, *I-TAC* IFN-γ T-cell Attractant Chemokine, *MIG* Monokine Induced by IFN- γ; *MCP* Monocyte Chemotactic Protein, *IP-10* IFN- γ Inducible Protein-10

### Whole blood cultures show that *C. burnetii* induces production of CXCR3-chemokines

**Fig. 2:**
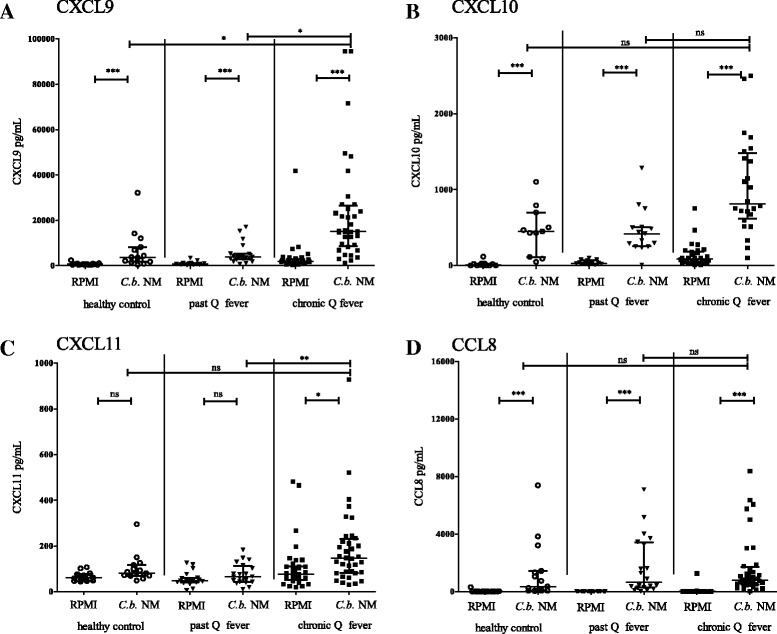
Chemokine production in whole blood cultures. **a**–**d** Chemokine production in whole blood cultures of healthy controls (*n* = 15), past Q fever infected patients (*n* = 20) and chronic Q fever patients (*n* = 36) after 24 h stimulation with C. burnetii 10^7^/mL compared to culture medium RPMI. Medians with IQR are indicated. Kruskal Wallis with Dunn’s multiple comparisons test was performed. *: *p* < 0.05, **: *p* < 0.01, ***: *P* < 0.001, ns: not significant. Abbreviations: RPMI, Roswell Park Memorial Institute Medium; Cb NM, C. burnetii Nine Mile, IQR, Interquartile range

To validate the results from the gene expression analysis and to assess the suitability of measurement of the gene-products, the production of these proteins in *C. burnetii* stimulated whole blood was determined (Fig. 2). These experiments were performed with blood from 15 healthy volunteers, 20 past Q fever and 36 chronic Q fever patients, who were diagnosed (median) 20 months before blood sampling.

Healthy controls showed significant *C. burnetii-*induced production of CXCL9, CXCL10 and CCL8, but no production of CXCL11. The same pattern was seen in past Q fever individuals. Chronic Q fever patients showed increased production of all four chemokines (CXCL9, CXCL10, CXCL11 and CCL8). CXCL9 production was significantly higher in chronic Q fever patients (median 15.1 ng/mL, IQR 8.5–26.5) than in healthy controls (median 3.6 ng/mL, IQR 1.7–8.2, *p* < 0.05) and past Q fever patients (median 3.8 ng/mL, IQR 2.8–5.3, *p* < 0.05). CXCL11 production in chronic Q fever patients (median 146 pg/mL, IQR 83–230) was higher than in past Q fever patients (median 66 pg/mL, IQR 43–183, *p* < 0.05).

A Receiver-operator-curve (ROC) analysis was performed for CXCL9 and CXCL11 to distinguish between chronic Q fever patients and past Q fever patients. The test accuracy, denoted by the area under the curve (AUC), was 85% (CI: 75–95%) for CXCL9 and 78% (CI: 66–90%) for CXCL11.

Further analysis of the CXCL11 production in chronic Q fever patients showed that production in samples taken within 3 months after diagnosis (*n* = 5) was significantly higher than in samples taken later in the course of the disease (*n* = 20, *p* < 0.05), but not for the other chemokines.

Because the identified chemokines are all induced by IFN-γ, their correlation with *C. burnetii*-specific IFN-γ production in the whole blood culture supernatants was assessed. Generally, correlation was moderate (Additional file 1: Fig. S1). The correlation coefficient (Spearman) was 0.54 for CXCL9 (*p* < 0.001), 0.70 for CXCL10 (*p* < 0.001), 0.50 for CXCL11 (*p* < 0.001) and 0.53 for CCL8 (*p* < 0.001).

### Circulating CXCL9 serum levels are high in chronic Q fever patients

We measured circulating chemokines in serum of 9 healthy controls, 10 past Q fever individuals, 9 acute Q fever and 51 chronic Q fever patients. Chronic Q fever patients were treated for a median of 13 months (range 0–46).

**Fig. 3:**
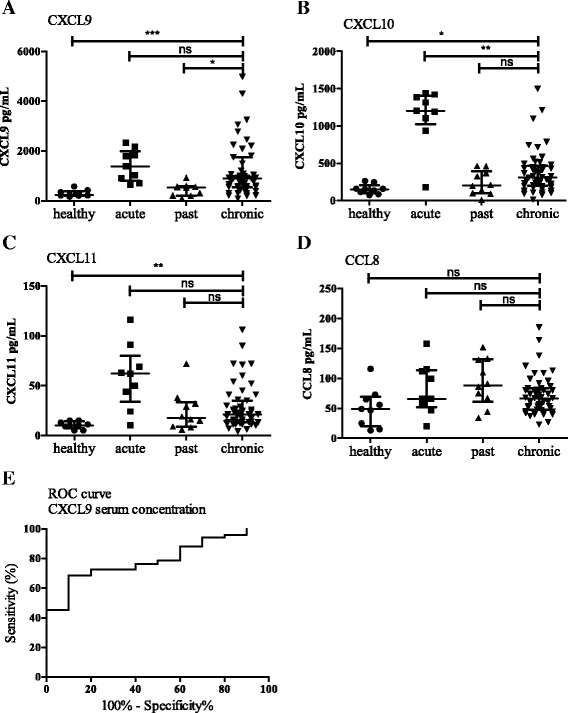
Circulating chemokines in serum. Circulating levels of chemokines, medians with interquartile range are indicated. Panel (**a**–**d**) display CXCL9, CXLC10, CXCL11 and CCL8 serum levels respectively, in healthy controls (*n* = 9), past Q fever individuals (*n* = 10), acute Q fever (*n* = 9), and chronic Q fever (*n* = 51). Kruskal Wallis with Dunn’s multiple comparisons test was performed. Panel (**e**) represents the Receiver Operating Characteristic (ROC curve) of CXCL9 concentration in serum in chronic Q fever patients or past Q fever individuals. *: *p* < 0.05, **: *p* < 0.01, ***: *P* < 0.001, ns: not significant

Chronic Q fever patients exhibited significantly higher serum concentrations of CXCL9 (median 899 pg/mL, range 92–12,734), CXCL10 (median 311 pg/mL, range 16–1497) and CXCL11 (median 21 pg/mL, 4–106) compared to healthy controls (CXCL9 230 pg/mL, 164–557, *p* < 0.001, CXCL10 151 pg/mL, 73–264, *p* < 0.05, CXCL11 10 pg/mL, 5–15, *p* < 0.01, Fig. 3). Chronic Q fever patients showed higher CXCL9 serum concentrations than past Q fever individuals (median 537 pg/mL, 82–927). Chronic Q fever patients did not have higher levels of CXCL10 or CXCL11 than past Q fever individuals (median 204 pg/mL,10–464 and median 18 pg/mL, 6–72). Concentrations of CCL8 were low in all groups and did not differ between groups. As compared to chronic Q fever patients, acute Q fever patients had higher CXCL10 serum concentrations (median 1204 pg/mL, 180–1439, *p* < 0.01). The other chemokines did not differ between chronic Q fever and acute Q fever patients.

ROC analysis of the CXCL9 serum concentrations of chronic Q fever compared to past Q fever individuals resulted in a test accuracy of 79% (denoted by the AUC, CI 67–92%, Fig. 3e). The sensitivity was 73% and specificity 80%, when a cut-off (580 pg/mL) was chosen that yielded both a high sensitivity and specificity.

Clinical parameters of the patients at diagnosis, such as PCR positivity in blood or tissue, and anti *C. burnetii* phase I IgG titers were compared to the performance of CXCL9 serum concentration at the 580 pg/mL cut-off value (Table 2 and a detailed description in Additional file 2: Table S1).

**Table 2:** Comparison of diagnostic characteristics and CXCL9 serum concentration

Diagnostic parameters	Proven chronic Q fever (*n* = 38), (%)	Probable chronic Q fever (*n* = 13), (%)
IgG phase *I* > 1:1024 at diagnosis	32/32 (100%)	11/13 (85%)^a^
PCR blood/tissue positive	26/31 (81%)	-
CRP >10 mg/L at diagnosis	5/14 (36%)	1/2 (50%)
Serum CXCL9 > 580 pg/mL	31/38 (82%)	6/13 (46%)

^a^Two patients had IgG phase I levels of 1: 512 and were diagnosed based on FDG-positive lesions on the ventral side of the heart with a mechanical aortic valve and a FDG-avid lesion in the ascending aorta, near the pre-existing aneurysm of the thoracic aorta

Further analysis of the CXCR3-chemokine serum concentrations revealed that samples taken within one month after diagnosis of chronic Q fever (*n* = 9) were significantly higher for CXCL9 (*p* < 0.05) and CXCL10 (*p* < 0.05) than samples taken more than 1 month after diagnosis (*n* = 41)*.*

### Circulating serum concentrations of CXCR3-chemokines during follow-up show no correlation with treatment duration

**Fig. 4:**
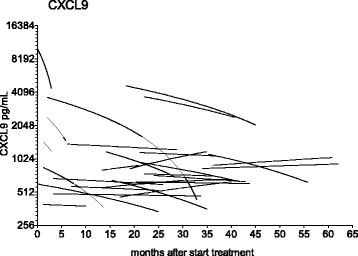
Non linear regression lines of CXCL9 serum concentrations per patient. Non-linear regression lines of CXCL9 serum concentrations per chronic Q fever patient in months from diagnosis, a best fit model was adapted

As circulating CXCR3-ligands were high in chronic Q fever patients, we investigated whether they would decrease during the course of treatment and could be used as marker for monitoring the effect of treatment. To this end, we analysed serum samples taken at regular intervals of 28 chronic Q fever patients during their visits to the outpatient clinic. In total, 137 serum samples were available (median 5 samples per patient (range 2–7), between 0 to 62 months after start of treatment). In a nonlinear best fit regression model, the slopes of the regression lines per patient were calculated and the median slope was generated for CXCL9 (−5.0, range − 1959 to 23), CXCL10 (−0.8, range − 125,550 to 22), CXCL11 (−0.2, −14 to 1.6) and CCL8 (−0, −19 to 35) indicating that there was no relation between chemokine levels and treatment duration per patient (Fig. 4). Similar results were obtained when time after last positive PCR in blood was taken into account.

### CXCR3-chemokines are weakly correlated to IgG phase I and C-reactive protein

In 135 serum samples from 40 chronic Q fever patients the corresponding anti-*C. burnetii* IgG phase I titers were known. There was a weak correlation between IgG phase I titers and CXCL9 (Spearman *r* = 0.24, *p* < 0.01) but no correlation with the other chemokines.

The acute phase protein C-reactive protein is elevated in various inflammatory conditions including Q fever and is determined during treatment to monitor disease activity. Seventy-six CRP values were available from 27 patients. Fifty-eight samples were below the detection limit of the test (5 mg/L), whereas only 5 CRP values were above 50 mg/L. We assessed the correlation of the CRP values to the concurrent chemokine serum concentrations and found a weak correlation with CXCL9 (*r* = 0.25, *p* < 0.05) and CXCL11 (*r* = 0.47, *p* < 0.001), but not with the other chemokines.

## Discussion

In the present study, we searched for new biomarkers for diagnosis and monitoring of chronic Q fever. With a transcriptome analysis, we found 4 highly up-regulated chemokine genes, induced by IFN-γ, in response to heat-killed *C. burnetii*. Subsequent whole blood stimulation experiments and the measurement of serum concentrations showed that *C. burnetii-*stimulated CXCL9 and CXCL11 production was higher in chronic Q fever patients compared to past Q fever individuals, and that serum concentrations of CXCL9 were higher in chronic Q fever patients compared to past Q fever individuals. This suggests that particularly CXCL9 could aid in the diagnosis of chronic Q fever.

CXCL chemokines are small chemotactic proteins, characterized by the presence of four cysteines with a variable amino acid ‘X’ dividing them [13]. As suggested by their alternative names, the four mentioned chemokines share the same inducer IFN-γ. In addition, CXCL10, CXCL11 and CCL8 can also be induced by type I interferons and by TNF-α, whereas this is not the case for CXCL9. These chemokines can be expressed by a variety of cells such as PBMCs, macrophages, endothelial cells and fibroblasts [13]. CXCL9, CXCL10 and CXCL11 are a ligand of the same CXCR3-receptor located on NK-cells, effector T-cells, dendritic cells and many non-immune cell types. Even though these 3 chemokines have similar properties, their affinity for CXCR3 varies and their expression depends on time and stimulus, suggesting critical differences in production and function [14, 15]. CXCL9 and CXLC10 are degraded by matrix metalloproteinase-8 (MMP-8) and MMP-9 respectively [16], therefore, differences in concentration of active MMP-8 or MMP-9 may influence the levels and functionality of CXCL9 and CXCL10. With respect to the immune response against intracellular bacteria such as *C. burnetii*, it is of interest that CXCR3-chemokines have also been examined as candidate markers for the differentiation between active and latent tuberculosis [17], as a possible alternative for IFN-γ release assay (IGRA) in latent tuberculosis [18] and for follow-up of tuberculosis treatment [19].

As we published recently, *C. burnetii* antigen-specific CXCL10 production was high during the acute stage of *C. burnetii-*infected BALB/c mice [20]. In line with this, we report high CXCL10 serum concentrations in human acute Q fever patients during the early stage of their disease. Although it is tempting to speculate that CXCL10 may be an early marker for acute Q fever, CXCL10 is likely an inflammation marker rather than being specific for acute Q fever [21, 22].

Previously, we published that cytokine production (TNF-α, IL-1β, IL-1Ra, IL-4, IL-5, IL-6, IL-10, IL-12p70, IL-23, IL-18) in *C. burnetii*-stimulated diluted blood did not distinguish seropositive controls from chronic Q fever patients, whereas the ratio of IFN-γ/IL-2 production appeared to accurately identify chronic Q fever patients with 79% sensitivity and 96% specificity [23]. Despite its usefulness, this assay is rather laborious, because it demands stimulation and incubation of whole blood cultures. Reviewing the results from the current study we can argue that simple measurement of CXCL9 serum concentration may also be of help in distinguishing chronic Q fever from past infection, although its specificity and sensitivity is somewhat lower than the IFN-γ/IL-2 production assay.

It is reasonable to believe that the circulating CXCR3 ligands are not exclusively raised in chronic Q fever. Circulating CXCL9 and CXCL10 concentrations are also elevated in patients with heart failure, but to a lesser extent than what we observed in chronic Q fever patients [24, 25]. More importantly, we have not addressed the specificity of CXCL9 in relation to other pathogens that elicit a T-helper 1 cell response, such as mycobacterium tuberculosis and others. It is likely that these infections will at least temporarily induce elevated serum concentrations of the CXCR3 ligands. These caveats need to be considered when measuring CXCL9 serum concentrations in patients suspected of a chronic infection. *C. burnetii* stimulated CXCL9 production may be more specific than serum concentration, although this was not tested in the current study. It has to be noted that conclusions on CXCR3 biomarkers should not be based on the transcriptome analysis alone, but need to be considered in light of the validation experiments. Additionally, the small sample size of the transcriptome analysis deems cautious interpretation.

Another limitation is the variable time after diagnosis of chronic Q fever at which the first sample was taken, 9 serum samples were taken within one month after diagnosis. This may have led to an underestimation of the discriminative power in the early phase of the disease. Adding to this effect is the unknown Q fever status of part of the healthy controls who provided samples for whole blood stimulation. In the Netherlands, within the center of the endemic area, the prevalence of *C. burnetii* antibodies is about 5% [26].

We also focused on the capacity of IFN-γ induced CXCR3-chemokines to monitor disease activity in chronic Q fever. Overall, we found that serum samples taken shortly after diagnosis displayed higher CXCL9 and CXCL10 concentrations compared to samples drawn after long-term antibiotic treatment. Assuming that antibiotics are effective in lowering disease activity, this may indicate that CXCL9 and CXCL10 levels are related to disease activity. However, we found no correlation between chemokine levels and duration of antibiotic treatment. Of note, all chronic Q fever patients included in the present study were still under treatment and according to the traditional markers of disease activity, were considered to have active disease. The absence of cured patients in our cohort limited our ability to detect any direct correlation between chemokine levels and disease activity.

Traditional markers for diagnosing and monitoring chronic Q fever are anti-phase I IgG titers, reflecting a specific B-cell derived humoral immune response and CRP, a product of an aspecific IL-6 mediated inflammatory response, used to detect complications and concomitant infections. Arguably, these markers might not accurately reflect disease activity, since the protective immune response against *C. burnetii* is predominantly an IFN-γ mediated T-helper 1 response. Anti-phase I IgG antibodies play a relatively small role in the adequate immune response to *C. burnetii* [27, 28].

Reportedly, CRP is high in the acute stage of acute Q fever [29, 30]. However, as we have shown, CRP is undetectable in most samples of chronic Q fever patients and therefore not a suitable marker for disease remission in chronic Q fever. Thus, the weak correlation of CRP and CXCL9 found in our study is expected. Given the small role of the humoral immune response in intracellular infections, it may not be surprising that serum CXCL9 is only weakly correlated with anti-phase I IgG titers.

## Conclusion

In conclusion, CXCL9, as a marker of cell-mediated immunity, is a promising candidate to improve the diagnostic accuracy of chronic Q fever.

## Additional files

Additional file 1: Fig. S1. Correlation of chemokine production with interferon-γ production. Panel A–D: Scatter plots of chemokine production and corresponding interferon-γ production after ex-vivo whole blood stimulation with heat killed C. *burnetii* NM 10^7^/mL. (EPS 134 kb)

Additional file 2: Table S1. Patient Characteristics. Diagnostic characteristics of the chronic Q fever patients cohort. (PDF 37 kb)

## Abbreviations

AUC: Area Under Curve
*C. burnetii*: *Coxiella burnetii*
CRP: C-reactive protein
CWZ hospital: Canisius Wilhelmina Hospital
CXCR3: CXC3-Receptor
ELISA: Enzyme-linked immunosorbent assay
FDG-PET/CT: Fluorodeoxyglucose-Positron Emission Tomography/Computer Tomography
IFA: Immunofluorescence assay
IFN-γ: Interferon-gamma
IGRA: Inteferon-γ release assay
IL: Interleukin
IP-10: IFN-γ Inducible Protein-10
IQR: Interquartile range
I-TAC: IFN-γ T-cell Attractant Chemokine
MCP-2: Monocyte Chemotactic Protein-2
MIAME: Minimun Information About a Microarray Experiment
MIG: Monokine Induced by IFN-γ
NK-cells: Natural Killer cells
NM: Nine Mile
PBMC: Peripheral blood mononuclear cell
PCR: Polymerase Chain Reaction
RNA: Ribo nucleic acid
ROC: Receiver operator characteristic
TNF-α: Tumor Necrosis Factor-α
