# Supplementary material for: CXCL9, a promising biomarker in the diagnosis of chronic Q fever
Source: BMC Infect Dis. 2017 Aug 9;17:556. doi: 10.1186/s12879-017-2656-6 (PMC5551022; doi:10.1186/s12879-017-2656-6)
Supplement: Supplementary file 2 — Patient Characteristics. Diagnostic characteristics of the chronic Q fever patients cohort. (PDF 37 kb) [file 12879_2017_2656_MOESM2_ESM.pdf]

Supplementary Table 1 Diagnostic parameters of the chronic Q fever patient cohort

Patient	Chronic Q fever diagnosis	PCR blood/tissue	IgG phase I at diagnosis	CRP at diagnosis (mg/L)	CXCL9 (pg/ml)
1	proven	positive	32768	5	12734
2	proven	positive	2048	unknown/nd	10170
3	proven	positive	4096	unknown/nd	4978
4	proven	positive	131072	22	4295
5	proven	positive	unknown	unknown/nd	3250
6	proven	unknown	32768	unknown/nd	3052
7	proven	positive	8192	unknown/nd	2464
8	proven	unknown	2048	unknown/nd	2248
9	proven	positive	32768	15	2206
10	proven	positive	8192	unknown/nd	2088
11	proven	positive	32768	5	1808
12	proven	negative	65536	58	1496
13	proven	unknown	1024	unknown/nd	1215
14	proven	negative	65536	unknown/nd	1080
15	proven	positive	4096	unknown/nd	1056
16	proven	positive	4096	unknown/nd	1038
17	proven	negative	1024	34	990
18	proven	positive	unknown	unknown/nd	930
19	proven	positive	1024	unknown/nd	926
20	proven	positive	8192	6	900
21	proven	negative	unknown	93	886
22	proven	unknown	2048	unknown/nd	837
23	proven	unknown	unknown	unknown/nd	824
24	proven	positive	8192	unknown/nd	782
25	proven	positive	4096	unknown/nd	775
26	proven	positive	4096	unknown/nd	724
27	proven	positive	unknown	unknown/nd	698
28	proven	positive	32768	5	672
29	proven	positive	65536	5	612
30	proven	positive	4096	unknown/nd	589
31	proven	positive	8192	unknown/nd	583
32	proven	positive	unknown	5	542
33	proven	unknown	32768	unknown/nd	484
34	proven	positive	131072	5	394
35	proven	positive	8192	5	307
36	proven	positive	2048	unknown/nd	213
37	proven	negative	8096	unknown/nd	207
38	proven	negative	1024	5	92
39	probable	unknown	1024	unknown/nd	2778
40	probable	negative	4096	unknown/nd	1741
41	probable	unknown	16384	30	1009
42	probable	negative	2048	unknown/nd	934

43	probable	negative	512	unknown/nd	929
44	probable	negative	512	unknown/nd	899
45	probable	unknown	16384	unknown/nd	553
46	probable	negative	4096	unknown/nd	550
47	probable	negative	32768	unknown/nd	443
48	probable	negative	4096	5	409
49	probable	negative	8192	unknown/nd	297
50	probable	unknown	8192	unknown/nd	295
51	probable	unknown	4096	unknown/nd	191

Supplementary table 1. Diagnostic characteristics of chronic Q fever patient cohort. Abbreviations: unknown/nd: unknown or not determined.
